# Progress in Gynæcology

**Published:** 1902-09-13

**Authors:** 


### Progress in Gynæcology.

**Retroflexion of the Uterus.**—J. Bland-Sutton[1] strongly advocates the operation of hysteropexy for the cure of retroflexion of the uterus, and thinks that treatment by pessaries is useless, and in many cases harmful. He believes that retroflexion giving rise to severe symptoms is usually complicated with the presence of small ovarian cysts, prolapsed ovaries, and chronically-inflamed tubes, and that one advantage of the treatment of these cases by hysteropexy is, that any abnormal condition of the tubes or ovaries can be dealt with at the same time. He emphasises the importance of care in the selection of cases for this operation, and considers there is one class of patients in whom it is strongly contra-indicated and that is in "alcoholics." Le Roy Broun[2] thinks that Alexander's operation is pre-eminently the safest and best method to restore and retain an uncomplicated retro-displaced uterus in its forward position. He reports 230 cases with two deaths, neither of these, however, were attributable to the operation. In none of his cases was there any complication during subsequent pregnancy or labour. Thomas G. Stevens[3] considers that cases of retroflexion in which the uterus is freely movable, and the ovaries not tender, should be treated by replacing the uterus, and keeping it in position by means of a well-fitting pessary. In cases where the fundus is fixed by adhesions, and the displacement cannot be reduced, he thinks that vaginal cœliotomy is the best procedure. The adhesions should be carefully broken down, the ovaries and tubes inspected and treated if necessary, and the uterus fixed in an anteverted position to the vaginal wall. He prefers this method to Alexander's operation, and to ventrofixation. J. M. Balby[4] recommends the following operation for these cases. The peritoneum is opened, the round ligament on each side picked up and divided close to the uterus. A pair of forceps is then made to perforate the broad ligament from its posterior aspect; the cut end of the round ligament is grasped in the bite of the forceps, and pulled through the hole in the broad ligament until it protrudes on its posterior aspect. The opposite side is then treated in a similar manner. The cut ends of the round ligaments are then attached to the posterior aspect of the uterus near the cornua, as much of the ligament being cut off as is necessary to bring the uterus into its proper position. The advantages claimed for this operation are, that the uterus is free to expand during pregnancy, and there are no adhesions present that may give rise to future trouble from pain, or possible strangulation of a loop of intestine. B. H. Wells,[5] as the result of his own experience, lays down the following rules with regard to the various abdominal operations that are performed for retroflexion of the uterus:—(1) Intra-abdominal shortening of the round ligaments should be done in cases in which it is necessary to open the abdomen for the treatment of complicating conditions, and in cases of persistent retroflexion in which the displacement interferes with health and cannot be relieved by simpler measures. (2) Ventral suspension is indicated in old women in whom there is no risk of a subsequent pregnancy. (3) Intra-abdominal shortening of the round ligaments should be the operation of choice in young women in whom there is a probability of future conception. H. N. Vineberg,[6] writing on this subject, advocates vaginal suturing of the round ligaments, and reports 50 cases without a death. In 47 of these the operation resulted in an anatomical success, and there had been four pregnancies terminating normally at full term.

**Appendicitis in Connection with Pelvic Disease.**—Fraenkel[7] points out the importance of recognising that pelvic disease may be secondary to lesions of the appendix. Infection may extend to the appendix from the diseased pelvic organs, or vice versa, in two ways. (1) By an intra-peritoneal route along the blood and lymph vessels of the suspensory ligament of the ovary, or by direct contact of an abnormally long appendix with the uterus or right tube and ovary. (2) By an extra-peritoneal route between the folds of the mesocæcum, or in consequence of the extra-peritoneal development of the cæcum and appendix. The most frequent pelvic complication of appendicitis is suppurative inflammation of the tubes and ovaries, especially on the right side. In every case of appendicitis in the female a thorough examination of the pelvis should be made. The appendix should also be examined in every case of abdominal section for pelvic disease, and should be removed not only when it is manifestly diseased, but even when it is adherent, since if it be spared the adhesions generally re-form, and morbid changes are apt to occur in the muscular wall of the appendix in consequence.

1 Clin. Jour., May 28, 1902. 2 Med. Rec., Feb. 22, 1902. 3 Treatment, Feb., 1902. 4 Med. Prog., June 7, 1902. 5 Med. Rec., June 28, 1902. 6 Ibid. 7 Amer. Jour. of Med. Sci., March, 1902.

(*To be continued.*)

